# Associations Between Socioeconomic Factors and Alcohol Outcomes

**DOI:** 10.35946/arcr.v38.1.11

**Published:** 2016

**Authors:** Susan E. Collins

**Affiliations:** Susan E. Collins, Ph.D., is an associate professor in the Department of Psychiatry and Behavioral Sciences, University of Washington, Harborview Medical Center, Seattle, Washington

**Keywords:** Alcohol consumption, alcohol-related problems, alcohol-related consequences, special populations, socioeconomic status, socioeconomic factors, economic disparities, racial minority, ethnic minority, homeless

## Abstract

Socioeconomic status (SES) is one of the many factors influencing a person’s alcohol use and related outcomes. Findings have indicated that people with higher SES may consume similar or greater amounts of alcohol compared with people with lower SES, although the latter group seems to bear a disproportionate burden of negative alcohol-related consequences. These associations are further complicated by a variety of moderating factors, such as race, ethnicity, and gender. Thus, among individuals with lower SES, members of further marginalized communities, such as racial and ethnic minorities and homeless individuals, experience greater alcohol-related consequences. Future studies are needed to more fully explore the underlying mechanisms of the relationship between SES and alcohol outcomes. This knowledge should be applied toward the development of multilevel interventions that address not only individual-level risks but also economic disparities that have precipitated and maintained a disproportionate level of alcohol-related consequences among more marginalized and vulnerable populations.

According to the World Health Organization (2014), alcohol consumption is responsible for approximately 5.9 percent of deaths worldwide and a global loss of 139 million disability-adjusted life-years. The alcohol-related disease burden is precipitated in part by acute intoxication, which decreases reaction time, perception and motor skills, and inhibitions and is thereby associated with an increased risk for traffic accidents, self-inflicted injuries, suicide, falls, drownings, alcohol poisoning, and interpersonal violence. Longer-term effects of alcohol consumption also contribute to the disease burden by way of various medical conditions (e.g., cancer, cardiovascular disease, and liver cirrhosis) and psychiatric disorders (e.g., depression and alcohol use disorder [AUD]). Given the strong positive association between alcohol use and negative alcohol-related consequences, it is important to understand social determinants of these alcohol outcomes.

The quantity and frequency of a person’s alcohol use, the resulting negative alcohol-related consequences (also known as alcohol-related problems), and his or her risk of AUD are determined by a variety of influences. These include higher-level chrono- and macrolevel factors, such as historical time and geopolitical context, as well as meso-, micro-, and individual-level factors, such as community context, family/peer influences, biological predisposition, effects of prenatal alcohol exposure, psychological factors, and sociodemographic features (e.g., gender, age, race, ethnicity, culture, religious affiliation, and socioeconomic status [SES]) (Edwards 2000; Gately 2008). These factors, which operate within various systems and levels, interact and transact over time to determine alcohol-related outcomes, such as drinking patterns and negative alcohol-related consequences (Gruenewald et al. 2014; Holder 1998).

This article focuses on one particular aspect of this complex set of systems, namely the relationship between SES—including income/economic factors, educational level, employment status, and housing status—and alcohol-related outcomes. It synthesizes data primarily obtained from English-language systematic reviews and meta-analyses that were based on studies conducted in the past decade involving adult populations (for a summary of these reviews and meta-analyses, see table 1). In some cases, these analyses were limited to studies from only one country, whereas other analyses were cross-national. In any case, caution must be used when interpreting these findings, because the cultural and political contexts in which these phenomena occur can differ widely. In addition, this article reviews some larger, population-based studies (see table 2), particularly those that were not addressed within the included reviews and which directly assess the association between SES and alcohol consumption and related outcomes. Although most of the studies only included adults, a few also involved adolescents when meta-analyses and reviews did not exclude such studies.

Across the studies discussed in this article, SES has been operationalized on various levels (e.g., individual, area/neighborhood, and national levels) using a variety of parameters, such as personal income and debt, family or household income, educational level, employment status, and housing status; neighborhood or area disadvantage; and gross national income. Although these variables often are interrelated, this article addresses economic, income, and educational factors; employment status; and housing status in separate sections to facilitate interpretation of the overall findings.

Alcohol-related variables evaluated in this article, which were assessed either cross-sectionally or longitudinally, include the following:

- Alcohol use, which is operationalized either continuously (e.g., by quantity and/or frequency of alcohol use or heavy episodic drinking [HED],^1^ defined as consuming four or more drinks per episode for women and five or more drinks per episode for men), or dichotomously by alcohol-use status (e.g., ever-drinker, heavy drinker, heavy episodic drinker);
- Presence of AUD; and
- Alcohol-related problems, including alcohol-related mortality.

It is important to keep in mind that these are outcomes at the individual level; however, alcohol use and misuse certainly also have consequences at the familial, community, or societal levels. A discussion of these consequences is outside of the scope of this article.

The article first summarizes cross-sectional perspectives on the associations of socioeconomic variables such as income, economic factors, and educational level with the quantity and frequency of alcohol use as well as negative alcohol-related consequences. In addition, it reviews the findings of longitudinal analyses regarding the associations between SES and alcohol-related outcomes before focusing on studies assessing two specific socioeconomic variables—i.e., employment status and housing—and their relationship with alcohol outcomes and touching on the effects of changes in SES on alcohol use and its consequences. A discussion of the limitations of the existing research and future directions concludes the review. Note that in some of the studies discussed, alcohol-related variables have been collapsed with other drug-related variables (e.g., any alcohol or other drug [AOD] use, alcohol and nicotine dependence), and this is noted accordingly.

## Cross-Sectional Associations Between SES Variables and Alcohol Outcomes

### Quantity and Frequency of Alcohol Use

In the past decade, several population-based studies, but no meta-analyses or systematic reviews, have assessed the cross-sectional relationship between snapshots of SES and quantity and/or frequency of alcohol use. These studies typically have focused on either individual-level (e.g., personal income, debt, or education) or area-level (e.g., neighborhood median income or economic disparities in a given region) SES variables.

The Centers for Disease Control and Prevention (CDC) (2012) conducted a population-based study of the association between HED and several SES-related variables among adults (*N* = 457,677) in 48 States and Washington, DC. The findings indicated that people who did not graduate from high school and had a low income had the lowest prevalence of HED. In fact, HED prevalence increased with household income and was highest among those with a household income greater than $75,000 a year. However, among those respondents who did engage in HED, those who reported the lowest educational and income levels reported the highest frequency of HED and the highest quantity consumed per occasion (CDC 2012). Another population-based study conducted in New York City at the neighborhood level yielded similar findings (Galea et al. 2007). Specifically, the neighborhoods with the highest income and with the greatest income disparities showed the highest prevalence of alcohol use as well as greater frequency of drinking. Similarly, analysis of data from a large, population-based survey called the Panel Study of Income Dynamics demonstrated that three indicators of family-background SES—income, wealth, and parental education—predicted alcohol use in young adults (Patrick et al. 2012). Young adults with the highest family-background SES reported greater alcohol use, and those with greater family wealth reported higher monthly HED prevalence. It is conceivable, however, that other factors, such as regional differences or personal characteristics (e.g., religiosity) may influence these associations.

A few studies have examined alternative operationalizations of individual-level SES by looking at each participant’s subjective assessment of his or her social status (Finch et al. 2013) or personal unsecured debt (Richardson et al. 2013). Finch and colleagues (2013) found that subjective social status was not associated with level of alcohol use; however, consistent with the findings of other studies, personal and household income were positively correlated with alcohol-use quantity and frequency as well as frequency of HED. Richardson and colleagues (2013) conducted a meta-analysis of 65 studies examining the effects of personal, unsecured debt on various health outcomes, including 5 studies that included alcohol-related outcomes. The findings from those studies indicated that personal, unsecured debt was associated with 2.68 times higher odds of “problem drinking,” which was variously defined as higher quantity/frequency of alcohol use, HED, or presence of AUD.

In another review of 41 studies, Karriker-Jaffe (2011) examined whether area-level disadvantage (i.e., the effects of living in a certain neighborhood, zone, county, or country) was associated with increased AOD use. The studies included in the analysis assessed the impact of a wide range of area-level SES effects. The review concluded that residents in a given area were relatively similar in their AOD use (i.e., AOD-use outcomes clustered by geographic area). However, the studies reviewed provided only limited and conflicting support for the hypothesis that area-level disadvantage was associated with increased AOD use, with some effects supporting the hypothesis and others pointing in the opposite direction (i.e., indicating that area affluence was associated with increased alcohol use). A wide range of factors related to the populations studied (e.g., age and ethnicity), the size of the areas examined, the specific SES measures used, the specific outcomes evaluated, and the analytic techniques employed all seemed to influence the association between SES and AOD use. Similarly, in a review of 48 studies, Bryden and colleagues (2013) reported inconclusive findings regarding the association between alcohol use and various measures of SES (e.g., neighborhood deprivation, poverty, income levels, and unemployment). The analyses did, however, offer area-level corroboration of the conclusions from individual-level studies because there was some indication that adults living in higher-income areas reported greater alcohol use. The findings also indicated a protective effect of the level of community participation and involvement on alcohol use.

Another population-based study (Karriker-Jaffe et al. 2012) that used data from the 2000 U.S. Census and the 2000 and 2005 National Alcohol Surveys (NAS) (*N* = 13,864) examined relationships between neighborhood disadvantage (i.e., low levels of education, employment, and income/financial assets) and several parameters, including levels of abstinence, heavy drinking, and negative alcohol-related consequences. Analyses using various models incorporating both individual-level and neighborhood-level measures indicated that individual-level SES had the strongest impact on drinking patterns and consequences. When such individual-level factors were removed from the models, neighborhoods with lower SES were characterized by greater prevalence of alcohol abstinence compared with neighborhoods with higher SES, although among those who did drink, neighborhood disadvantage was associated with heavy drinking and negative alcohol-related consequences. These associations were moderated by various demographic characteristics, such as race/ethnicity and gender. Thus, African-American and Hispanic men were excluded from the protective effect of neighborhood disadvantage on risk of any drinking. Furthermore, neighborhood disadvantage was associated with reduced heavy drinking for European Americans but with increased heavy drinking for African Americans.

To some extent the racial/ethnic differences may be the result of different levels of exposure to social disadvantage. Thus, in a separate analysis of data from the 2005 NAS (Mulia et al. 2008) that compared the relationship among social disadvantage, stress, and alcohol use among Black, Hispanic, and White Americans, the investigators found that for all three racial/ethnic groups, exposure to social disadvantage (e.g., greater poverty, unfair treatment, racial or ethnic stigma) was associated with problem drinking. However, Blacks and Hispanics reported greater exposure to social disadvantage than Whites, which may account for higher rates of problem drinking.

Additional analyses of data from the 2000 U.S. Census and 2000 and 2005 NAS (Mulia and Karriker-Jaffe 2012) further identified interactions between individual-level and neighborhood SES that influenced alcohol consumption and related problems. Among men, living in a neighborhood with higher SES was associated with higher odds of heavy drinking and intoxication only among those with a low individual SES compared with men with a middle or higher SES living in the same advantaged neighborhoods. In contrast, neighborhood disadvantage was associated with an increased risk for alcohol-related problems in women, and individual-level SES did not seem to influence this association.

### Alcohol-Related Harm and AUD

Studies have shown a strong association between SES and alcohol-related mortality, the most severe form of alcohol-related harm. In a meta-analysis of 15 studies capturing data on approximately 133 million people worldwide, Probst and colleagues (2014) examined the association between SES (operationalized as a pooled measure reflecting occupation, employment status, income, and education) and alcohol-related mortality as well as all-cause mortality. The analyses found that lower SES increased the risk of alcohol-related mortality by 66 percent for men and 78 percent for women compared with all-cause mortality.

Additional studies have supported these findings. In a recent study involving data from the U.S. Health and Retirement survey (*N* = 8,037), being in the most disadvantaged SES quartile was associated with a 2.84 times greater risk of all-cause mortality than being in the most advantaged quartile. Mediating factors, including alcohol use, smoking, and physical inactivity, significantly and collectively accounted for 68 percent of this all-cause mortality (Nandi et al. 2014). Further, a Finnish study of men ages 25–64 showed that individual-level socioeconomic (i.e., higher education and occupation status) and cultural (i.e., being part of the Swedish-speaking minority) factors were protective against alcohol-related mortality. As with the association with alcohol use discussed earlier, these factors typically dwarfed the influence of area-level factors (Blomgren et al. 2004). Thus, neither area-level median income nor income inequality was associated with alcohol-related mortality. Nevertheless, some area-level SES variables (i.e., percentage of manual laborers and unemployment) were significant risk factors for alcohol-related mortality when explored on their own.

Other investigators have focused on negative alcohol-related consequences beyond mortality. A meta-analysis of cross-sectional surveys conducted across 25 countries (*N* = 42,655) indicated that men and women with less education were more likely to report negative alcohol-related consequences than their more educated counterparts—even after controlling for drinking patterns (Gritt– ner et al. 2012). In addition, men from countries with lower gross national incomes reported more societal consequences of drinking compared with men from countries with higher gross national incomes (Grittner et al. 2012). Again, these effects of SES-related variables on negative alcohol-related consequences may be moderated by other individual-level factors, such as race and ethnicity. A recent population-based study in the United States (*N* = 13,997) that explored socioeconomic disparity by race and ethnicity (Karriker-Jaffe et al. 2013) determined that in States with greater between-race income inequality, African-American and Latino/Hispanic individuals were at greater risk for negative alcohol-related consequences and alcohol dependence than were European-American individuals.

Finally, Lee and colleagues (2013) evaluated the relationship between SES and AUD in a study (*N* = 808) of substance-use (i.e., alcohol, nicotine, and cannabis) and psychiatric-disorder (i.e., depression and anxiety) latent classes. The study identified four groups of participants: those with virtually no symptoms of mental health or substance-use problems, those with symptoms of licit-substance use disorders (mostly alcohol and nicotine dependence), those with mental health disorder symptoms, and those with comorbid symptoms of all five mental health and substance-use indicators. The analysis suggested that the relationship between SES and AUD is not simply unidirectional but that effects actually occur in both directions. Thus, the investigators found that people who did not earn their high school diploma by age 21 were more than twice as likely to belong to the alcohol- and nicotine-dependence group and six times more likely to belong to the comorbid-symptoms group compared with those who had achieved a higher educational attainment. At the same time, people with greater alcohol- and nicotine-dependence symptoms or comorbid symptomatology achieved lower wealth accumulation at age 30 compared with people with low overall symptom experience (Lee et al. 2013). Taken together, these findings indicate a strong, bidirectional relationship between SES and alcohol-related harm. Specifically, people with lower SES tend to experience more negative alcohol-related consequences than people with higher SES. Further, people with greater experience of negative alcohol-related consequences tend to have lower income.

## Longitudinal Associations Between SES and Alcohol Outcomes

Looking beyond static and cross-sectional relationships of SES and alcohol use and its consequences is important for understanding developmental changes in alcohol-related variables as a function of changing SES and vice versa. These associations have been studied using a variety of strategies. A few studies have examined the relationship between childhood SES and later alcohol use and related outcomes, often without identifying a clear association. For example, a systematic review of 19 international longitudinal studies of childhood SES and alcohol use in adulthood only revealed weak and inconsistent associations between childhood SES and later drinking (Wiles et al. 2007). Another 25-year longitudinal study that followed African-American children through young adulthood (*N* = 1,242) found no significant direct effects of childhood SES (i.e., parental education and family income) on later AOD problems (Fothergill and Ensminger 2006). However, the study did identify significant indirect effects of lower SES, such that lower SES predicted fewer years of education, which in turn increased the risk for AOD problems.

Poonawalla and colleagues (2014) used a different approach by conceptualizing SES not as static but as a trajectory of its own. Using latent-class growth analysis of data from the Study of Early Child Care and Youth Development survey (*N* = 1,356 families), these investigators examined the relationship between childhood SES trajectories and alcohol-use prevalence at age 15. The analyses indicated that family-level economic downturns predicted past-year drinking at age 15. Similarly, a French occupational cohort study (*N* = 20,570) suggested that downward or steadily disadvantaged SES trajectories along with alcohol and tobacco use predicted greater later all-cause mortality (Melchior et al. 2006).

A third approach used in longitudinal analyses is to follow the alcohol trajectories of participants and relate these to SES. Such studies have yielded mixed findings. Platt and colleagues (2010) focused on U.S. adults over age 50, assessing their alcohol use as well as a variety of demographic, socioeconomic, and other characteristics. The study found that alcohol use generally tended to decrease over time in this population. However, the investigators identified a minority (2.2 percent) of individuals with increasing alcohol use. This group was largely characterized by greater affluence, European-American race, male gender, nonmarried status, lower levels of religiosity, and good-to-excellent health, thus suggesting that increased alcohol use was associated with higher SES. Conversely, a Finnish study following participants (*N* = 1,334) from ninth grade through adulthood found that people with increasing and heavy-drinking trajectories from ages 16 through 42 had greater socioeconomic difficulties at age 42, even after controlling for baseline SES (Berg et al. 2013).

## Associations Between Specific Socioeconomic Variables and Alcohol Use

### Employment Status

Compared with various measures of SES discussed in many of the above studies (e.g., neighborhood disadvantage, personal income, household income, and education), the association of employment status with alcohol use is less equivocal. Thus, a systematic review of five studies suggested that adult unemployment was associated with increased levels of alcohol use (Bryden et al. 2013). It should be noted, however, that the review included only a relatively small number of studies and that those studies primarily involved adolescents.

A few population-based studies have corroborated these findings. Popovici and French (2013) conducted a fixed-effect analysis of data from waves 1 and 2 of the population-based National Epidemiologic Survey of Alcohol and Related Consequences (NESARC) (*N* = 43,093). The investigators found that past-year unemployment was associated with increases in average daily alcohol quantity, HED frequency, and probability of an AUD diagnosis. Compton and colleagues (2014) analyzed the associations between unemployment and heavy drinking and AUD using data from the U.S. National Survey on Drug Use and Health between 2002 and 2010, taking into consideration the economic downturn during that time period. The analyses indicated that unemployment was significantly associated with heavier alcohol use and AUD and that this association was nearly independent of gender, age, or race/ethnicity. This association did not significantly differ between the periods before and after the economic downturn of 2008.

### Housing Status

Homelessness may be viewed as an extreme form of socioeconomic disadvantage and marginalization.^2^ The top reasons for homelessness include lack of sufficient income, loss of employment, and increased expenses, as well as lack of affordable housing (Mojtabai 2005; Tessler et al. 2001).

In addition to socioeconomic disadvantage, homeless individuals are disproportionately affected by other problems. For example, the prevalence of alcohol use among homeless individuals has been estimated to be as high as 80 percent (Velasquez et al. 2000), which is substantially higher than in the general population. A meta-analysis of international studies determined a mean alcohol-dependence prevalence of 38 percent among homeless individuals (Fazel et al. 2008), which is 10 times the prevalence of alcohol dependence in the general U.S. population (Grant et al. 2004). Chronically homeless people also often have severe and persistent psychiatric, medical, and substance-use disorders (Collins et al. 2012; Fazel et al. 2008; Hwang 2001; Mackelprang et al. 2014; Martens 2001). Together, these factors lead to greater mortality, including increased alcohol-related mortality, in the homeless population (Hawke et al. 2007; Hwang et al. 2009; O’Connell 2005) as well as an increased burden on the health care and criminal justice systems (Larimer et al. 2009; World Health Organization 2011).

Several studies have suggested that housing status and alcohol outcomes may share a complex longitudinal association that is apparent across the lifespan. For example, a study of 370 adolescents indicated that recent homelessness was the strongest predictor of subsequent substance abuse (Tompsett et al. 2013). In addition, a within-subject analysis involving the older and more severely affected end of the homeless population (i.e., chronically homeless individuals with alcohol dependence) showed that alcohol use and negative alcohol-related consequences seemed to decrease as a function of time spent in housing (Collins et al. 2012). Thus, homelessness seems to precipitate substance abuse, and the provision of adequate and low-barrier housing to people affected by homelessness may in turn reduce negative alcohol-related consequences.

## Effects of Changes in SES on Alcohol Use and Its Consequences

As indicated previously, not only overall SES but also changes in SES may have an impact on people’s alcohol use and its consequences. The economic recession that affected the United States between 2007 and 2009^3^ has afforded researchers an opportunity to study the consequences of such economic downturns. Mulia and colleagues (2014) used data from the 2009–2010 NAS (*N* = 5,382) to assess the association between economic loss and alcohol consumption, intoxication, negative alcohol-related consequences, and alcohol dependence. The analyses found that severe economic loss, such as loss of a job or housing, was associated with greater experience of negative alcohol-related consequences, alcohol dependence, and intoxication, whereas moderate economic loss, such as loss of retirement savings or reduced work hours or wages, had no such impact.

Several sociodemographic characteristics, such as gender, age, and race/ethnicity, moderated these associations. For example, women affected by economic loss showed increased alcohol consumption, whereas men showed increased intoxication, drinking consequences, and alcohol dependence (Mulia et al. 2014). Additional analyses of the same dataset determined that the association between exposure to severe economic loss and alcohol consumption and related consequences differed among Blacks, Hispanics, and Whites. Thus, not only were Blacks and Hispanics more likely than Whites to experience economic loss, such as job loss or housing problems, but Blacks also had a significantly higher risk than Whites of experiencing two or more negative alcohol-related consequences and alcohol dependence when experiencing severe economic loss (Zemore et al. 2013). For Hispanics, in contrast, only weak and ambiguous associations existed between economic loss and alcohol outcomes.

Other less concrete factors, such as informal social support systems, also may influence the association between changes in SES and alcohol use and alcohol-related negative consequences. When researchers examined the effects of housing instability (e.g., difficulties paying rent or mortgage as well as loss of housing) on alcohol use during the 2007–2009 recession, they confirmed the findings described earlier that housing instability was associated with more negative alcohol-related consequences and increased risk of alcohol dependence (Murphy et al. 2014). This association was modified by perceived family support—that is, respondents who thought that they had greater support from their families reported fewer alcohol-related consequences compared with respondents with less perceived support. These observations further underscore that the relationships between SES and alcohol use and related consequences are highly complex and influenced by a multitude of interacting factors.

## Limitations

The existing research reviewed here has some important limitations that deserve mention. First, some of these meta-analyses, reviews, and studies have conflated measures of alcohol use (e.g., quantity/frequency measures) with measures of negative alcohol-related consequences. For example, in their analysis, Richardson and colleagues (2013) combined higher levels of alcohol use (i.e., greater quantity and HED frequency) with AUD symptomatology into one construct of “problem drinking,” even though none of the studies they included in their meta-analysis used designated measures of negative alcohol-related consequences. Future research should more clearly differentiate between these measures and terms to avoid confusion, because heavier drinking does not necessarily translate into a greater experience of negative alcohol-related consequences or problem drinking.

Second, relatively few meta-analyses have comprehensively explored the associations between various conceptualizations of SES and alcohol outcomes. Therefore, the current overview and many of the reviews cited within rely on subjective assessments of the literature. Given the number of studies that have been conducted in this area, this approach is an inefficient way to synthesize such a complex body of research (Borenstein et al. 2009). Therefore, future research should involve more comprehensive meta-analyses to more rigorously analyze the association between SES and various operationalizations of alcohol use and related outcomes (e.g., quantity/frequency, experience of negative alcohol-related consequences, and presence of AUD). Such meta-analyses also should consider the moderation of these associations by other factors, such as race, ethnicity, gender, housing status, or drinking status. A more comprehensive approach would help better understand the relationship between SES and alcohol outcomes and their repercussions for more marginalized groups in our society.

## Summary and Future Directions

This review has summarized the current state of knowledge regarding the associations between SES and alcohol use and its negative consequences, based on a variety of study approaches (e.g., cross-sectional vs. longitudinal studies, meta-analyses vs. summary reviews, population-based vs. individual-level studies). The literature on the cross-sectional associations between alcohol use and individual- and area-level income and economic factors mostly has supported a positive relationship between SES and alcohol use, such that individuals with higher SES (or living in areas with higher SES) engage in more frequent and heavier drinking. However, this relationship may be moderated by other individual-level variables, such as drinking status, gender, race, and ethnicity (CDC 2012; Karriker-Jaffe et al. 2012). Therefore, future studies should clarify these associations by simultaneously examining the roles of these factors, particularly within meta-analyses that could capitalize on increased power to identify significant moderating effects.

In contrast to the findings for alcohol use, cross-sectional analyses have indicated that SES is inversely related to negative alcohol-related consequences, including alcohol-related mortality. In other words, although people with lower SES may be less likely to drink and may be consuming less alcohol overall, they are more negatively affected by its effects. Findings to date suggest that economic disparities and their secondary effects are moderating the relationship between alcohol use and the experience of negative alcohol-related consequences; however, the exact nature of these complex relationships requires further exploration.

Research on the long-term associations between SES and alcohol outcomes has shown inconsistent correlations between snapshots of childhood SES and later alcohol outcomes. In contrast, a relatively consistent, inverse association seems to exist between long-term trajectories of SES and alcohol outcomes, with downward SES trajectories predicting heavier subsequent drinking and greater negative alcohol-related consequences. Further studies involving more sophisticated longitudinal analytic methods (e.g., cross-lagged panel modeling) are needed to more explicitly test and establish the nature of the complex transactional dependencies between the trajectories of SES and alcohol outcomes over time.

Two of the numerous factors that can be used to operationalize and assess SES are employment and housing status, and the relationship of these two factors with alcohol use and related outcomes sometimes has been evaluated separately from more general SES studies. Such studies have indicated that among adults, unemployment is associated with increased drinking and elevated risk for AUD. Interestingly, this relationship has not seemed to be affected by the economic downturn in 2008 (Compton et al. 2014). Taking a cue from the longitudinal literature discussed above, however, future studies should focus on evaluating the effects of changing employment status on alcohol outcomes and negative alcohol-related consequences.

Although homelessness may be considered a more extreme form of socioeconomic disadvantage, its effects on individuals go beyond those of SES. The literature on housing status and alcohol outcomes shows an unequivocal and clinically significant association between homelessness and increases in alcohol use, negative alcohol-related consequences, and AUD prevalence. In recent years, research efforts have begun to shed light on the relationship between homelessness and alcohol outcomes (U.S. Department of Health and Human Services 2007). However, more research is necessary to fully assess and address the needs of this marginalized population, which is multiply affected by psychiatric, medical, and substance-use disorders and disproportionately uses high-cost health care and criminal justice services.

Taken together, the findings discussed in this review suggest that although individuals with higher SES may consume similar or greater amounts of alcohol compared with individuals with lower SES, the latter group seems to bear a disproportionate burden of negative alcohol-related consequences. Future studies—particularly rigorous meta-analyses—are needed to more fully explore the mechanisms underlying these relationships. This research can contribute to data gathered in the context of larger public health efforts, including the Healthy People 2020 Initiative, which seeks to assess health disparities in the U.S. population by tracking rates of death, chronic and acute conditions, and health-related behaviors for various marginalized subpopulations (U.S. Department of Health and Human Services 2010). This knowledge should be applied toward the development of multilevel interventions that address not only individual-level risks but also economic disparities at higher levels that have precipitated and maintained a disproportionate level of negative alcohol-related consequences among more marginalized and vulnerable populations. Such interventions would fit well in the context of larger public health efforts (e.g., Affordable Care Act; HHS Action Plan to Reduce Racial and Ethnic Health Disparities) that are aiming to increase access to health care among people with low SES, create more preventative health programs, and improve quality of care for people seeking health care services in lower-SES areas (U.S. Department of Health and Human Services 2010, 2011).

## Figures and Tables

**Table 1:** Summary of Meta-Analyses and Reviews of Cross-National Studies Reporting on the Association Between Socioeconomic Status (SES) and Alcohol Outcomes

Authors	Type	Number of Studies Included	Variables Analyzed	Main Findings Regarding the Association Between SES and Alcohol Outcomes
Bryden et al. 2013	Systematic review	48	Association between community-level social factors and alcohol use among adults and adolescents	Findings were inconclusive for associations between alcohol use and deprivation, poverty, income, unemployment, social disorder, and crime. Social-capital characteristics (e.g., social support, community cohesion, social participation, supportiveness) may protect against alcohol use.
Fazel et al. 2008	Meta-analysis	29 (*n* = 5,684)	Prevalence of psychiatric disorders among homeless people	Prevalence of psychiatric disorders varied greatly among studies. The most common psychiatric disorders were alcohol dependence (prevalence 8.1 to 58.5 percent) and drug dependence (prevalence 4.5 to 54.2 percent).
Grittner et al. 2012	Meta-analysis	Survey data from 42,655 individuals in 25 countries participating in the Gender, Alcohol and Culture: An International Study (GENACIS)	Association of country-level characteristics and individual SES and individual alcohol-related consequences	Lower gross national income was associated with more social problems in men. Lower educational attainment was associated with more reported alcohol-related consequences at comparable drinking levels in both men and women.
Karriker-Jaffe 2011	Systematic review	41; 34 studies used for main analysis	Association between area-level disadvantage and substance use	Strong evidence suggested that substance-use outcomes cluster by geographic area. There was limited/conflicting support that area-level disadvantage is associated with increased substance use. The association between area-level disadvantage and substance use seemed to vary according to age, ethnicity, size of area examined, type of SES measure, specific outcome analyzed, and analysis techniques.
Probst et al. 2014	Meta-analysis	15	Association between SES and alcohol-related mortality vs. all-cause mortality	For both men and women, lower SES was associated with 1.5- to 2-times-higher alcohol-related mortality compared with all-cause mortality. Alcohol consumption and SES interacted to lead to greater harm in people with lower SES even at comparable levels of alcohol consumption.
Richardson et al. 2013	Meta-analysis	65, including 5 studies (*n* = 26,706) assessing problem drinking	Association between personal, unsecured debt and health outcomes (eg, various mental disorders, suicide attempt or completion, problem drinking, drug dependence)	Most studies found that more debt is related to worse health (i.e., increased odds of mental disorders, alcohol and drug dependence, suicide attempt or completion). A significant relationship existed between debt and problem drinking (odds ratio = 2.68).
Wiles et al. 2007	Systematic review	19 longitudinal studies	Association between childhood SES and alcohol use later in life	Evidence indicated only weak and inconsistent associations between lower childhood SES and later alcohol use and abuse.

**Table 2:** Summary of the Design and Main Findings of Population-Based Studies Concerning the Association Between Socioeconomic Status (SES) and Alcohol Outcomes

Authors	Type; Country of Study	Number of Participants	Variables Analyzed	Main Findings Regarding the Association Between SES and Alcohol Outcomes
Berg et al. 2013	Longitudinal; Finland	1,334	Association between drinking trajectories and adult health and socioeconomic disadvantage	Among Finnish men, those with a steady high or increasing drinking trajectory had an increased risk of experiencing health and economic disadvantage. Among Finnish women, those with a steady high drinking trajectory had an increased risk of almost all health and economic disadvantages.
Blomgren et al. 2004	Cross-sectional; Finland	1.1 million	Association between individual-level and area-level SES characteristics and alcohol-related mortality	Individual-level socioeconomic and cultural factors were protective against alcohol-related mortality. Some, but not all, area-level factors were protective against alcohol-related mortality. Individual-level SES factors had a greater impact than area-level factors.
Centers for Disease Control and Prevention 2012	Cross-sectional; United States	457,677	Prevalence, frequency, and intensity of heavy episodic drinking (HED) and influence of various sociodemographic variables	Overall prevalence of HED was 17.1 percent; among binge drinkers the average frequency was 4.4 episodes per month and the average intensity was 7.9 drinks per occasion. With respect to household income, binge-drinking prevalence was highest among those with the highest income (> $75,000), but frequency and intensity were highest among those with the lowest income (< $25,000).
Collins et al. 2012	Longitudinal; United States	95	Association between project-based Housing First and alcohol-use trajectories among homeless people	Time spent in low-barrier, non–abstinence-based, permanent, supportive housing (Housing First model) was associated with declining alcohol use. Greater number of months spent in housing predicted additional decreases in alcohol use.
Compton et al. 2014	Cross-sectional; United States	Ca. 405,000	Association between employment status and alcohol and other drug outcomes	Unemployment was associated with higher rates of heavy alcohol use, past-year alcohol and other drug abuse/dependence, and past-month tobacco and illicit drug use. Marked increases in unemployment rates during the recent recession did not moderate these associations.
Fothergill and Ensminger 2006	Longitudinal; United States	1,242	Association between childhood/adolescent antecedents and adult alcohol and drug problems in African Americans	Educational attainment was associated with reduced risk of substance-use problems.
Galea et al. 2007	Cross-sectional; United States	1,355	Association between neighborhood income and income distribution and prevalence and frequency of alcohol and other drug use	Neighborhoods with both the highest income and the highest income maldistribution had the highest prevalence of alcohol use. On an individual level, both high neighborhood income and income maldistribution were associated with greater likelihood of alcohol use as well as with greater frequency of alcohol use.
Karriker-Jaffe et al. 2012	Cross-sectional; United States	13,864	Association between neighborhood disadvantage and alcohol outcomes (drinking, heavy drinking, alcohol-related consequences, dependence)	Neighborhood disadvantage was significantly associated with increased abstinence among all groups except for African-American and Hispanic/Latino men. Neighborhood disadvantage was inversely associated with heavy drinking for White drinkers but positively associated with heavy drinking for African-American drinkers. Neighborhood disadvantage was marginally associated with elevated alcohol-related consequences among those who do drink, particularly among African-American men and White women.
Karriker-Jaffe et al. 2013	Cross-sectional; United States	13,997	Association between State-level income inequality (Black–White and Hispanic–White poverty ratios) and alcohol outcomes	Higher Black–White poverty ratios were associated with higher levels of light and heavy drinking among Whites and Blacks. Higher Black–White poverty ratios were associated with increased alcohol-related consequences and dependence for Blacks. Higher Hispanic–White poverty ratios were associated with higher levels of light drinking by Whites and Hispanics. Higher Hispanic–White poverty ratios were associated with increased alcohol-related consequences and dependence for Hispanics.
Melchior et al. 2006	Longitudinal; France	20,570	Association between socioeconomic trajectory and mortality	Steadily disadvantaged SES or downward SES trajectory increased risk of premature all-cause mortality. Alcohol consumption was one of the factors explaining this association.
Mulia and Karriker-Jaffe 2012	Cross-sectional; United States	8,728	Association between neighborhood and individual SES and alcohol use and alcohol-related problems	For men with low SES, living in a neighborhood with a high SES was associated with increased risk drinking, intoxication, and alcohol-related problems. For women, living in a neighborhood with low SES was associated with increased risk of alcohol problems, but no interactions existed with individual SES.
Mulia et al. 2008	Cross-sectional; United States	6,631	Association between social disadvantage (poverty level, frequency of unfair treatment, racial/ethnic stigma consciousness) and alcohol outcomes (drinking, at-risk drinking, problem drinking)	Blacks and Hispanics reported greater exposure to social disadvantage than Whites. In all groups, exposure to social disadvantage was associated with problem drinking. Frequent unfair treatment, high racial stigma, and extreme disadvantage was associated with 2 to 6 times greater experience of alcohol problems. The association can be partially explained by psychological distress.
Mulia et al. 2014	Cross-sectional; United States	5,382	Association between types of economic loss and alcohol outcomes	Severe economic loss (job, housing) was positively associated with negative drinking consequences, alcohol dependence, and, marginally, with intoxication. Moderate economic loss (retirement savings, reduced hours/wages, trouble paying bills) was unassociated with alcohol outcomes. Gender and age moderated these associations.
Murphy et al. 2014	Cross-sectional; United States	5,307	Association between housing instability and alcohol outcomes (social, legal, work-related, health, injuries/accidents) during the 2007–2009 U.S. recession	Both unstable and lost housing were associated with more alcohol problems and alcohol dependence symptoms. Perceived family support moderated the associations. Greater family support was associated with fewer alcohol problems, irrespective of housing instability. Job loss was not associated with alcohol outcomes if housing instability was included in the analysis.
Nandi et al. 2014	Cross-sectional; United States	8,037	Associations between SES, health behaviors (drinking, smoking, physical inactivity), and all-cause mortality	Being in the subpopulation with the lowest SES was associated with increased mortality. Drinking, smoking, and physical inactivity accounted for about two-thirds of the increased mortality risk.
Patrick et al. 2012	Cross-sectional; United States	1,203	Association between family SES (income, wealth, parental education) and substance use (drinking, smoking, marijuana use) in young adults	Alcohol and marijuana use in young adults were associated with higher family SES. HED in young adults was most strongly predicted by greater family wealth. Smoking in young adults was associated with lower family SES.
Platt et al. 2010	Longitudinal; United States	6,787	Association between drinking trajectories and various personal characteristics in older adults	Alcohol consumption declined for most adults studied, with substantial variation in the rate of decline; in a minority, alcohol consumption increased. High SES (affluence, high educational attainment) was associated with increasing alcohol consumption over time.
Poonawalla et al. 2014	Longitudinal; United States	1,356	Association of changes in family income with adolescent alcohol use and smoking	Family income trajectory was associated with past-year alcohol use at age 15 and ever-smoking at age 15. Children of families with declining SES were more likely to drink than were children from the most advantaged and most disadvantaged families.
Popovici and French 2013	Cross-sectional; United States	43,093	Association between employment status and alcohol outcomes	Job loss during the past year was positively associated with average daily alcohol consumption, frequency of HED, and alcohol abuse or dependence.
Tompsett et al. 2013	Longitudinal; United States	371	Association between substance abuse, affiliation with substance-using peers, and homelessness	Recent homelessness and affiliation with alcohol-using friends was associated with increased risk of alcohol abuse. The influence of alcohol-using friends on alcohol abuse decreased over time. The duration of initial homelessness did not influence substance abuse over time.
Zemore et al. 2013	Cross-sectional; United States	5,382	Associations among race/ethnicity, economic loss, and drinking	After experiencing severe economic loss, Blacks were more likely to experience alcohol-related problems and alcohol dependence compared with Whites. The associations between economic loss and alcohol outcomes were weak/ambiguous for Hispanics.

## Footnotes

1 The terms “heavy episodic drinking” and “binge drinking” have sometimes been used synonymously. The latter, however, has fallen out of favor with some alcohol researchers and treatment professionals because it can be confused with a longer-term and more extreme alcohol-use period than is typically referred to as a heavy drinking episode.

2 The U.S. Federal Government defines homelessness as lacking a fixed, regular, and adequate nighttime residence; having a primary nighttime dwelling that is not a regular sleeping accommodation; living in a supervised shelter or transitional housing; exiting an institution that served as temporary residence when the individual had previously resided in a shelter or place not meant for human habitation; or facing imminent loss of housing when no subsequent residence is identified and insufficient resources/support networks exist (Homeless Emergency and Rapid Transition to Housing [HEARTH] Act of 2009).

3 The National Bureau of Economic Research (2015) has officially dated the recession as lasting from December 2007 to July 2009; however, individual studies may refer to slightly different time periods.
